# Comparison of Amino Acid Digestibility between Commercial Crossbred Pigs and Mini-Jeju Island Native Pigs

**DOI:** 10.3390/ani14182687

**Published:** 2024-09-15

**Authors:** Hyunwoong Jo, John Kyaw Htoo, Beob Gyun Kim

**Affiliations:** 1 Department of Animal Science, Konkuk University, Seoul 05029, Republic of Korea; tazo88@konkuk.ac.kr; 2 Evonik Operations GmbH, Rodenbacher Chaussee 4, 63457 Hanau, Germany; john.htoo@evonik.com

**Keywords:** amino acid, mini-pig, prediction equation, standardized ileal digestibility, swine

## Abstract

**Simple Summary:**

There is a disadvantage in determining the amino acid digestibility of swine feed ingredients using commercial cannulated pigs due to their relatively fast growth rate. Using mini-pig breeds to determine the amino acid digestibility can complement this disadvantage of commercial pigs. Therefore, the objective of this study was to compare the ileal digestibility of crude protein and amino acids between commercial crossbred pigs and Jeju Island native pigs. The digestibility of most amino acids was comparable between the two breeds regardless of the test ingredient. These findings indicate that amino acid digestibility data from Jeju Island native pigs can be used to estimate the amino acid digestibility in feedstuffs for commercial crossbred pigs.

**Abstract:**

The objectives of this study were to determine the apparent ileal digestibility and standardized ileal digestibility (SID) of crude protein (CP) and amino acids (AA) in feed ingredients, compare the ileal digestibility of CP and AA between commercial crossbred pigs and mini-Jeju Island native pigs (JINP), and develop models for estimating SID of CP and AA for commercial pigs using mini-JINP data. The study involved five crossbred commercial pigs (31.5 ± 1.6 kg of body weight and 11 weeks of age; Landrace × Yorkshire) and five mini-JINP (31.0 ± 3.2 kg body weight and 20 weeks of age). The pigs were surgically equipped with a T-cannula at the end of ileum. Each pig breed was assigned to 5 dietary treatments in a 5 × 10 incomplete Latin square design with 10 periods. Four experimental diets were formulated to contain each of soybean meal, corn gluten feed, copra meal, and sesame expellers as the sole source of nitrogen. A nitrogen-free diet was also prepared to determine basal endogenous losses of CP and AA. No interaction between breed and feed ingredient was observed for the digestibility of CP and all indispensable AA. The SID of CP and all indispensable AA, except Arg, His, and Lys, did not differ between the two breeds of pigs. Prediction equations were developed for SID of CP and AA of commercial pigs using the SID values of mini-JINP: SID of CP (%) = (1.02 × SID of CP in mini-JINP) − 5.20 with r^2^ = 0.97 and *p* < 0.05; SID of Lys (%) = (1.12 × SID of Lys in mini-JINP) − 9.10 with r^2^ = 0.98 and *p* < 0.05; and SID of Met (%) = (1.08 × SID of Met in mini-JINP) − 4.27 with r^2^ = 0.96 and *p* < 0.05. The digestibility for most AA in feedstuffs for commercial pigs can be estimated using data from mini-JINP.

## 1. Introduction

Mini-pigs have been widely used as human models in various biomedical research areas, such as the development of new drugs, organ transplantation research for human organ replacement, biomedical technology development, and medical practice, due to their anatomical similarity to humans [1,2,3]. Furthermore, the smaller size and slower growth rate of mini-pigs compared to commercial pigs can be advantageous for metabolism studies [4]. The Jeju Special Self-Governing Province livestock promotion agency developed a laboratory mini-pig breed using Jeju Island native pigs (JINP) [5,6,7].

The estimation of ileal digestibility of amino acids (AA) can be conducted using both in vitro and in vivo methods [8,9,10]. In particular, estimating ileal digestibility through in vivo methods requires the attachment of a T-cannula to the distal ileum for sampling ileal digesta in pigs [11,12]. However, cannulated commercial pigs have disadvantages in general management and animal handling due to their relatively fast growth rate. As the mature size of the mini-pigs bred from JINP is much smaller compared with the commercial pigs, the mini-pigs equipped with ileal cannulas may last for a longer period for ileal digestibility assessments. If mini-pigs can be used for determining AA digestibility in the place of commercial pigs, the cost for cannulation surgery and animal handling will be largely saved. However, AA digestibility values in feed ingredients between mini-JINP and commercial pigs have not been compared. Therefore, this study aimed to measure ileal digestibility of crude protein (CP) and AA in feed ingredients and compare the digestibility values between commercial crossbred pigs and mini-JINP. Additionally, prediction models were developed for estimating standardized ileal digestibility (SID) of CP and AA for commercial pigs using mini-JINP data. For the wide range of applicability of the equations, four ingredients with varying AA digestibility values were used: soybean meal (SBM), corn gluten feed (CGF), copra meal (CM), and sesame expellers (SEP).

## 2. Materials and Methods

An AA digestibility experiment was performed in an environmentally controlled room at Konkuk University. The experimental protocol was approved by the Institutional Animal Care and Use Committee at Konkuk University (KU18126).

### 2.1. Experimental Diets and Ingredients

Four test ingredients, including SBM, CGF, CM, and SEP, were used (Table 1) to make experimental diets. Four experimental diets contained 30% of each test ingredient as the only source of AA (Table 2). Additionally, a nitrogen-free diet was formulated based mainly on cornstarch and sucrose to estimate the basal endogenous losses (BEL) of CP and AA. Chromic oxide at 0.5% was included as an indigestible index in all diets for the digestibility calculation. A vitamin-mineral premix was added into the diets to meet or exceed the nutrient requirements [13].

### 2.2. Animals and Experimental Design

A total of five 11-week-old commercial crossbred barrows (Landrace × Yorkshire) with an initial body weight (BW) of 31.5 ± 1.6 kg and five 20-week-old mini-JINP (M-Pig^®^; Cronex Co., Ltd., Hwaseong, Republic of Korea) with an initial BW of 31.0 ± 3.2 kg were fed 5 experimental diets in a 2 × 5 factorial treatment arrangement. All pigs were surgically fitted with a T-cannula at the end of ileum using the method described by Stein et al. [14]. All animals were raised in individual pens (1.2 m × 1.2 m) equipped with a feeder and a drinking nipple. Each pig breed was assigned to 5 dietary treatments in a 5 × 10 Latin square design with 10 periods, respectively. Potential residual effects were minimized using a spreadsheet-based program [15].

### 2.3. Feeding and Sample Collection

The daily feed allowance was calculated as 2.7 times the estimated maintenance requirement for energy (i.e., 197 kcal metabolizable energy/kg BW^0.6^; [13]) based on the BW of the pigs measured at the beginning of each period. The feed allowance was divided into 2 equal meals and given at 0830 and 1700 h. Each period consisted of a 5 d adaptation period and a 2 d collection period. Ileal digesta were collected from 0900 to 1700 h. For the collection of ileal digesta, a plastic sample bag with wire was fixed to the T-cannula. The plastic bag was checked at 30-min intervals and changed as needed. Immediately after collection, the ileal digesta were frozen at −20 °C.

### 2.4. Chemical Analyses

The ileal digesta samples were lyophilized using a freeze drier. Feed ingredients were analyzed for dry matter (method 930.15), CP (method 990.03), ether extract (method 920.39), ash (method 942.05), calcium (method 978.02), and phosphorus (method 946.06) as described in AOAC [16]. Neutral detergent fiber (NDF) and acid detergent fiber in the feed ingredients were also analyzed using an ANKOM 200 analyzer (A200 fiber analyzers, ANKOM technology, Macedon, NY, USA). Amino acids in the feed ingredients and ileal digesta were analyzed using ion-exchange chromatography with post-column derivatization with ninhydrin. Methionine and cysteine were oxidized with performic acid, which was neutralized with sodium metabisulfite [17,18]. Amino acids were liberated from the protein by hydrolysis with 6 *N* HCl for 24 h at 110 °C and quantified with the internal standard by measuring the absorption of reaction products with ninhydrin at 570 nm. Tryptophan was determined using high performance liquid chromatography with fluorescence detection (extinction 280 nm and emission 356 nm), after alkaline hydrolysis with barium hydroxide octahydrate for 20 h at 110 °C [19]. Chromium concentrations in experimental diets and ileal digesta were analyzed by using a UV/vis spectrophotometer (Optizen 2120UV, Mecasys Inc., Daejeon, Republic of Korea).

### 2.5. Calculations and Statistical Analyses

The calculated value for AA in experimental diets was used to determine the AA digestibility in the experimental diets using the index methods [20]. Data were statistically analyzed employing the MIXED procedure of SAS 9.4 (SAS Inst. Inc., Cary, NC, USA). Variation within treatment was calculated and outliers were identified using the UNIVARIATE procedure. Observations that deviated from the median by more than 3 times the interquartile range based on the SID of total indispensable AA in a test ingredient were identified as outliers. The data were analyzed as a 2 × 4 factorial arrangement with 2 breeds and 4 diets. The statistical model included breed, dietary treatment, and breed × dietary treatment as fixed variables, with the animal and period as random variables. The PDIFF option with Tukey’s adjustment was employed for pairwise comparisons among the least squares of means. A *t*-test was conducted using the TTEST procedure of SAS to compare the BEL of CP and AA between mini-JINP and commercial pigs. Using the PROC REG, prediction equations for AA digestibility of ingredients in commercial pigs were developed using AA digestibility of mini-JINP as an independent variable. The experimental unit was the pig, and statistical significance was declared at an alpha level of 0.05.

## 3. Results

During the experimental period, except for one mini-JINP that lost the T-cannula during the third period, all animals were in a good condition.

### 3.1. Nutrient Composition of Ingredients

The CP concentration in SBM, CGF, CM, and SEP was 47.9, 19.0, 21.4, and 41.2% as-is basis, respectively (Table 1). The ether extract concentration ranged from 1.05% in SBM to 18.2% in SEP. Copra meal had the greatest NDF and acid detergent fiber and SBM had the lowest fiber concentration. The Lys concentration in test ingredients ranged from 0.50% for CM to 2.86% for SBM.

### 3.2. Ileal Digestibility and BEL of CP and AA

No interaction between pig breed and feed ingredient was observed for apparent ileal digestibility (AID) of CP and indispensable AA (Table 3). The AID of all indispensable AA except Arg, His, and Val did not differ between mini-JINP and commercial breed. Regardless of the pig breed, SBM had the greatest AID of CP and indispensable AA (*p* < 0.001). In both mini-JINP and commercial pigs, the AID of Arg, Ile, Lys, Phe, Thr, and Trp in CGF was less (*p* < 0.001) than that in SEP. The AID of CP, Arg, His, Lys, Thr, and Trp did not differ between CM and SEP in both pig breeds.

The BEL of CP in mini-JINP were not different from those in commercial pigs (Table 4). The BEL of Ile, Leu, Trp, and Val for mini-JINP were greater (*p* < 0.05) than those of commercial pigs, but the BEL of other AA was not different between the breeds.

No interaction between pig breed and feed ingredient was observed for SID of CP and indispensable AA (Table 5). The SID of CP and all indispensable AA except Arg, His, and Lys did not differ between mini-JINP and commercial breed. Regardless of the pig breed, SID of CP and most AA in SBM was the greatest (*p* < 0.001) among the ingredients. In both mini-JINP and commercial pigs, the SID of Ile, Leu, Met, Phe, and Val in CGF and SEP was less (*p* < 0.001) than that in CM. The SID of all indispensable AA except Leu and Trp was not different between CGF and SEP.

### 3.3. Prediction Equations for Estimating SID of CP and AA

Prediction equations for estimating SID of CP and indispensable AA in commercial pigs were developed (Table 6): SID of CP (%) = 1.02 × SID of CP in mini-JINP (%) − 5.20 with r^2^ = 0.97 and *p* = 0.017; SID of Lys (%) = 1.12 × SID of Lys in mini-JINP (%) − 9.10 with r^2^ = 0.98 and *p* = 0.010; SID of Met (%) = 1.08 × SID of Met in mini-JINP (%) − 4.27 with r^2^ = 0.96 and *p* = 0.020; SID of Thr (%) = 0.75 × SID of Thr (%) in mini-JINP + 18.07 with r^2^ = 0.83 and *p* = 0.092; SID of Trp (%) = 0.87 × SID of Trp in mini-JINP (%) + 11.44 with r^2^ = 0.96 and *p* = 0.022.

## 4. Discussions

As laboratory animals, mini-pigs have been used due to their slow growth rate and small adult size [21,22]. Recently, the mini-JINP was developed by the Jeju Special Self-Governing Province livestock promotion agency in Korea for laboratory usage [5,7]. The mini-JINP can serve as an experimental animal for evaluating AA digestibility because the rapid growth rate of commercial pigs poses challenges for conducting longer experiments and handling heavier pigs during the experimental periods. However, potential differences in AA digestibility between mini-JINP and commercial pigs may exist if the two breeds have distinct digestive functions. Furthermore, the differences in AA digestibility between the two breeds may vary depending on the types of ingredients. Therefore, it is essential to compare AA digestibility of various feed ingredients between mini-JINP and commercial pigs. The present study employed 4 plant-based feed ingredients with varying protein and fiber concentrations to identify the differences in AA digestibility between the two breeds and investigate the relationships between AA digestibility values in mini-JINP and commercial pigs.

The concentrations of CP and most AA in SBM and CM were within the range of previously reported values [12,13,23,24]. In contrast, the CP and most AA concentrations of CGF and SEP used in this study were less than those reported in previous studies [12,24,25] but were similar to those found by Sauvant et al. [26]. The difference in nutrient compositions among studies may be due to various factors including varieties, origins, and processing procedures [27].

The differences in the ileal digestibility of most AA between mini-JINP and commercial pigs were not observed in the present work, which is in agreement with a previous study that reported no difference in AID of the 5 most-limiting AA between Gottingen mini-pigs and Saddleback pigs [28]. The similar ileal AA digestibility between two breeds may be attributed to their shared anatomical features and nutritional physiology. In a previous experiment, however, the AID of Lys, Leu, Ile, Phe, and Val of Duroc × Berkshire × Jiaxing crossbred barrows was approximately 3–6 percentage unit greater than that in commercial 3-way crossbred barrow (Duroc × Landrace × Yorkshire) [29]. Moreover, Cheng et al. [30] reported that the Xiangcun black pig had a greater apparent total tract digestibility of nutrients and better feed efficiency compared with Duroc pigs. Therefore, although no difference in ileal AA digestibility between mini-JINP and commercial pigs was found in the present study, the capacity of nutrient digestion and absorption differs depending on the pig breeds.

Regardless of the pig breed, the SID of CP and most indispensable AA in SBM, CM, and SEP used in the present study was within the range of previously reported values [12,13,24,25,26,31,32,33,34]. In contrast, the SID of CP and most indispensable AA in CGF was less compared with the value in previous studies [12,25]. The fiber concentration in CGF used in the present work did not largely differ from that in the previous studies [12,25]. The reason for the low digestibility of AA in CGF used in this work remains unclear. One possible explanation is that excessive heat treatment during the manufacturing process for starch production may have affected the AA digestibility in CGF. Heating is known to enhance the nutritional value of cereal grains by deactivating anti-nutritional factors [35] and denaturing the native protein structure in the meal [36]. However, excessive heat can adversely affect nutrient stability, particularly Lys, because the Maillard reaction may occur when ingredients containing AA and reducing sugars are exposed to heat and moisture [37]. Oliveira et al. [38] reported that the AA digestibility in SBM was reduced when autoclaved at 150 °C compared with 110 °C. Similarly, Almeida et al. [39] found a quadratic decrease in the AA digestibility in canola meal with increasing autoclaving time. Therefore, it is possible that the CGF may have undergone excessive heat treatment during the manufacturing process. Further research is warranted to investigate the AA digestibility in various CGF sources processed under different conditions. Both breeds showed the greatest digestibility of most AA in SBM among test ingredients, likely due to its lower NDF content [40]. This finding was consistent with previous studies that have reported lower digestibility of CP and AA with increasing NDF concentration [12,24,40].

The negative or very low values for AID of AA are mainly due to the contribution of endogenous losses of AA to the ileal digesta of pigs. The influence of endogenous losses of AA on AID values is enlarged particularly when the quantity of AA intake is small [41], such as with CGF in the present study, resulting in negative AID values of some AA in CGF. The low AID values for Gly and Pro are mainly due to the large BEL of Gly and Pro that are observed in the present work and previous studies [12,24,42,43]. The large BEL of Gly and Pro are most likely due to the abundancy of Gly and Pro in mucin, which is highly resistant to proteolysis and often reaches the end of the ileum.

The BEL of CP and indispensable AA for commercial pigs in the present study were fairly close to the calculated BEL based on a meta-analysis [43,44]. Basal endogenous losses of AA in pigs can be influenced by several factors including BW, types and concentrations of dietary fiber, feed intake, and ingredient composition [45]. However, these factors were controlled in the present experiment. Therefore, the higher BEL of some AA in mini-JINP compared with those in commercial pigs may be attributed to differences in breeds. Basal endogenous losses of nitrogen include digestive enzymes, sloughed-off cells, and intestinal microbes; therefore, BEL of nitrogen may differ depending on the breed of pig, likely due to different physiological characteristics. Bartelt et al. [46] also reported differences in the amounts of endogenous losses of nitrogen between Gottingen mini-pigs and Landrace pigs.

Because no interaction was observed between breed and feed ingredient, the prediction equations developed in the present work can rather precisely estimate the SID of AA in commercial pigs using the SID of AA in mini-JINP, as indicated by the high determination coefficients (r^2^ > 0.90). The strong relationships of AA digestibility across the two breeds suggest that mini-JINP can serve as an alternative animal for evaluating AA digestibility in ingredients fed to commercial pigs, offering great convenience in handling experimental animals and cost-effectiveness for researchers. Although the lack of interaction between pig breed and feed ingredient was apparent for SID of AA in SBM, CGF, CM, and SEP in the present study, this observation may not be applicable to other ingredients. Further research is warranted to test the interaction between breed and ingredient and validate the equations using different ingredients.

## 5. Conclusions

No difference was observed in the digestibility of most AA between the two breeds. Furthermore, the ranking of AA digestibility among ingredients was similar for both mini-JINP and commercial pigs. These findings indicate that the SID of AA determined in mini-JINP can be used to predict the SID of AA in commercial pigs for feed ingredients.

## Figures and Tables

**Table 1:** Analyzed chemical composition of feed ingredients (as-fed basis).

Item, %	Ingredient			
	Soybean Meal	Corn Gluten Feed	Copra Meal	Sesame Expellers
Dry matter	89.4	89.6	89.1	94.3
Crude protein	47.9	19.0	21.4	41.2
Ether extract	1.05	2.06	1.78	18.2
Ash	5.92	5.75	7.18	8.96
Calcium	0.26	0.11	0.23	1.07
Phosphorus	0.43	0.54	0.49	0.89
Neutral detergent fiber	6.23	32.8	55.5	35.7
Acid detergent fiber	3.20	9.05	31.5	19.5
Indispensable amino acids				
Arg	3.38	0.90	1.96	3.65
His	1.19	0.49	0.35	0.85
Ile	2.12	0.50	0.69	1.34
Leu	3.49	1.33	1.30	2.41
Lys	2.86	0.51	0.50	0.78
Met	0.62	0.26	0.28	0.93
Phe	2.32	0.53	0.86	1.72
Thr	1.76	0.61	0.61	1.11
Trp	0.64	0.09	0.17	0.49
Val	2.19	0.79	1.03	1.72
Dispensable amino acids				
Ala	1.99	1.41	0.89	1.77
Asp	5.18	0.99	1.62	2.85
Cys	0.69	0.41	0.29	0.32
Glu	8.18	2.68	3.66	6.89
Gly	2.48	1.62	0.81	1.49
Pro	2.27	0.70	0.84	1.36
Ser	1.99	1.41	0.89	1.77

**Table 2:** Ingredient and chemical composition of experimental diets (as-fed basis).

Item	Experimental Diet				
	Soybean Meal	Corn Gluten Feed	Copra Meal	Sesame Expellers	Nitrogen-Free
Ingredients, %					
Cornstarch	46.80	46.75	46.65	47.62	67.87
Soybean meal, 48% CP	30.00	-	-	-	-
Corn gluten feed, 19% CP	-	30.00	-	-	-
Copra meal, 21% CP	-	-	30.00	-	-
Sesame expellers, 41% CP	-	-	-	30.00	-
Sucrose	20.00	20.00	20.00	20.00	20.00
Soybean oil	-	-	-	-	4.00
Cellulose	-	-	-	-	4.00
Limestone	0.60	0.60	0.75	0.08	0.43
Dicalcium phosphate	1.40	1.45	1.40	1.10	2.00
Potassium carbonate	-	-	-	-	0.40
Magnesium oxide	-	-	-	-	0.10
Vitamin mix ^1^	0.10	0.10	0.10	0.10	0.10
Mineral mix ^2^	0.20	0.20	0.20	0.20	0.20
Sodium chloride	0.40	0.40	0.40	0.40	0.40
Chromic oxide	0.50	0.50	0.50	0.50	0.50
Analyzed composition, %					
Dry matter	92.4	92.1	92.3	91.4	92.5
CP	14.2	5.87	6.67	11.7	0.21
Ether extract	0.32	0.60	0.51	5.77	2.59
Ash	1.80	1.68	2.07	2.84	3.40

CP = crude protein. ^1^ Vitamin mix provided the following quantities per kg of complete diet: vitamin A, 20,000 IU; vitamin D_3_, 4000 IU; vitamin E, 180 IU; vitamin K, 2.5 mg; thiamin, 3.5 mg; riboflavin, 8.0 mg; pyridoxine, 5.0 mg; vitamin B_12_, 0.045 mg; pantothenic acid, 25 mg; folic acid, 2.5 mg; niacin, 42 mg; biotin, 0.2 mg. ^2^ Mineral mix provided the following quantities per kg of complete diet: Cu, 10 mg as copper sulfate; Fe, 100 mg as iron sulfate; I, 0.8 mg as calcium iodate; Mn, 60 mg as manganese sulfate; Se, 0.2 mg as sodium selenite; and Zn, 8 mg as zinc sulfate.

**Table 3:** Apparent ileal digestibility (%) of crude protein and amino acids (AA) in soybean meal (SBM), corn gluten feed (CGF), copra meal (CM), and sesame expellers (SEP) fed to mini-Jeju Island native pigs and commercial pigs.

Item	Mini-Jeju Island Native Pig				Commercial Pig				SEM	*p*-Value		
	SBM	CGF	CM	SEP	SBM	CGF	CM	SEP		Breed	Feed	Breed × Feed
*n*	9	5	7	7	9	6	6	7				
Crude protein	68.2 ^a^	18.1 ^bc^	28.3 ^bc^	32.0 ^bc^	66.9 ^a^	17.0 ^c^	27.5 ^bc^	33.8 ^b^	3.79	0.930	<0.001	0.942
Indispensable AA												
Arg	79.5 ^a^	30.8 ^cd^	63.5 ^ab^	60.2 ^ab^	77.9 ^a^	10.2 ^d^	54.8 ^bc^	51.8 ^bc^	4.75	0.016	<0.001	0.273
His	76.9 ^a^	42.4 ^b^	44.1 ^b^	44.9 ^b^	78.5 ^a^	40.5 ^b^	35.5 ^b^	38.3 ^b^	2.57	0.028	<0.001	0.112
Ile	76.3 ^a^	25.4 ^d^	54.0 ^b^	42.5 ^c^	80.0 ^a^	30.4 ^d^	55.7 ^b^	42.2 ^c^	2.28	0.229	<0.001	0.599
Leu	76.1 ^a^	48.7 ^c^	60.0 ^b^	46.0 ^c^	79.3 ^a^	51.6 ^c^	59.8 ^b^	44.0 ^c^	1.79	0.510	<0.001	0.309
Lys	78.1 ^a^	−14.1 ^cd^	1.2 ^bcd^	11.5 ^b^	80.4 ^a^	−15.7 ^d^	−7.8 ^bcd^	5.3 ^bc^	4.41	0.329	<0.001	0.464
Met	76.2 ^a^	41.9 ^e^	61.8 ^b^	52.9 ^cd^	80.1 ^a^	45.4 ^de^	61.4 ^bc^	51.0 ^de^	2.08	0.492	<0.001	0.341
Phe	74.8 ^a^	25.6 ^e^	60.6 ^b^	50.3 ^cd^	78.1 ^a^	26.5 ^e^	59.2 ^bc^	46.9 ^d^	2.40	0.933	<0.001	0.436
Thr	60.2 ^a^	−10.6 ^d^	22.8 ^bc^	20.8 ^bc^	66.1 ^a^	5.0 ^cd^	19.1 ^bc^	22.7 ^b^	4.24	0.176	<0.001	0.153
Trp	68.2 ^a^	−57.4 ^c^	21.6 ^b^	35.6 ^b^	74.5 ^a^	−44.7 ^c^	20.9 ^b^	37.0 ^b^	4.83	0.212	<0.001	0.548
Val	70.4 ^a^	26.6 ^d^	55.7 ^b^	41.8 ^c^	75.8 ^a^	34.9 ^cd^	57.4 ^b^	41.6 ^c^	2.22	0.077	<0.001	0.188
Dispensable AA												
Ala	62.6 ^a^	42.2 ^bc^	35.7 ^bc^	32.4 ^bc^	65.6 ^a^	44.4 ^b^	32.1 ^bc^	29.4 ^c^	3.62	0.917	<0.001	0.583
Asp	69.5 ^a^	−2.8 ^e^	39.7 ^b^	23.7 ^cd^	73.7 ^a^	2.9 ^e^	37.6 ^bc^	24.4 ^d^	3.04	0.411	<0.001	0.557
Cys	55.4 ^a^	23.9 ^bc^	14.8 ^c^	14.7 ^c^	61.5 ^a^	31.3 ^b^	13.9 ^c^	11.6 ^c^	3.72	0.331	<0.001	0.323
Glu	72.2 ^a^	46.4 ^b^	53.4 ^b^	46.0 ^b^	78.9 ^a^	45.6 ^b^	52.3 ^b^	44.8 ^b^	2.74	0.705	<0.001	0.239
Gly	30.9 ^ab^	36.8 ^ab^	−43.1 ^c^	0.2 ^bc^	72.8 ^a^	−48.4 ^c^	9.2 ^b^	−12.0 ^bc^	11.17	0.936	<0.001	<0.001
Pro	8.7 ^a^	−42.1 ^abc^	−283.5 ^bc^	−149 ^abc^	−106.1 ^ab^	−264.6 ^abc^	−622.8 ^d^	−329.3 ^c^	65.33	0.007	<0.001	0.172
Ser	68.7 ^a^	7.6 ^c^	35.9 ^b^	33.4 ^b^	71.7 ^a^	15.7 ^c^	32.3 ^b^	33.6 ^b^	3.02	0.461	<0.001	0.252

SEM = standard error of the means. ^a–e^ Least squares of means within a row without a common superscript letter are different (*p* < 0.05).

**Table 4:** Basal ileal endogenous losses (g/kg of dry matter intake) of crude protein and amino acids for mini-Jeju Island native pigs and commercial pigs ^1^.

Item	Mini-Jeju Island Native Pig	Commercial Pig	SEM	*p*-Value
Crude protein	26.6	24.4	4.31	0.715
Indispensable amino acids				
Arg	1.30	1.29	0.284	0.988
His	0.28	0.25	0.024	0.403
Ile	0.39	0.31	0.023	0.029
Leu	0.66	0.52	0.038	0.021
Lys	0.55	0.47	0.060	0.312
Met	0.14	0.11	0.010	0.067
Phe	0.47	0.39	0.028	0.064
Thr	0.81	0.68	0.053	0.110
Trp	0.20	0.16	0.011	0.027
Val	0.62	0.47	0.036	0.013
Dispensable amino acids				
Ala	1.03	0.88	0.155	0.513
Asp	1.18	0.98	0.082	0.164
Cys	0.31	0.26	0.020	0.143
Glu	1.42	1.19	0.118	0.255
Gly	2.92	2.75	0.521	0.812
Pro	10.3	12.0	2.584	0.675
Ser	0.90	0.73	0.084	0.212

SEM = standard error of the mean. ^1^ Each mean represents 9 observations for mini-Jeju Island native pigs and 7 observations for commercial pigs.

**Table 5:** Standardized ileal digestibility (%) of crude protein and amino acids (AA) in soybean meal (SBM), corn gluten feed (CGF), copra meal (CM), and sesame expellers (SEP) for mini-Jeju Island native pigs and commercial pigs.

Item	Mini-Jeju Island Native Pig				Commercial Pig				SEM	*p*-Value		
	SBM	CGF	CM	SEP	SBM	CGF	CM	SEP		Breed	Feed	Breed × Feed
*n*	9	5	7	7	9	6	6	7				
Crude protein	85.6 ^a^	60.2 ^b^	65.4 ^b^	53.1 ^b^	81.9 ^a^	53.4 ^b^	59.5 ^b^	52.0 ^b^	3.79	0.263	<0.001	0.774
Indispensable AA												
Arg	91.4 ^a^	75.5 ^abc^	83.9 ^ab^	71.2 ^bcd^	88.7 ^ab^	50.7 ^d^	73.3 ^abc^	61.8 ^cd^	4.75	0.006	<0.001	0.158
His	84.1 ^a^	60.0 ^bc^	68.8 ^b^	54.9 ^cd^	84.8 ^a^	56.1 ^cd^	57.4 ^cd^	47.2 ^d^	2.57	0.002	<0.001	0.055
Ile	82.0 ^a^	49.9 ^c^	71.5 ^b^	51.6 ^c^	84.6 ^a^	50.0 ^c^	69.6 ^b^	49.4 ^c^	2.28	0.861	<0.001	0.576
Leu	81.9 ^ab^	64.0 ^de^	75.5 ^bc^	54.5 ^f^	83.9 ^a^	63.6 ^e^	72.0 ^cd^	50.6 ^f^	1.79	0.339	<0.001	0.205
Lys	84.1 ^a^	19.7 ^bc^	35.5 ^b^	33.4 ^b^	85.2 ^a^	11.6 ^c^	20.0 ^bc^	23.1 ^bc^	4.41	0.045	<0.001	0.161
Met	83.1 ^a^	58.0 ^c^	77.3 ^ab^	57.4 ^c^	85.5 ^a^	58.1 ^c^	73.5 ^b^	54.6 ^c^	2.08	0.551	<0.001	0.329
Phe	81.0 ^a^	52.6 ^b^	77.3 ^a^	58.6 ^b^	83.2 ^a^	48.9 ^b^	73.1 ^a^	53.8 ^b^	2.40	0.198	<0.001	0.303
Thr	74.3 ^a^	29.9 ^d^	63.3 ^ab^	43.2 ^cd^	78.0 ^a^	39.1 ^cd^	53.1 ^bc^	41.5 ^cd^	4.24	0.939	<0.001	0.128
Trp	77.7 ^a^	12.8 ^c^	58.2 ^b^	47.9 ^b^	82.1 ^a^	11.7 ^c^	50.3 ^b^	46.9 ^b^	4.83	0.709	<0.001	0.572
Val	79.1 ^ab^	50.5 ^c^	74.2 ^ab^	52.8 ^c^	82.4 ^a^	53.1 ^c^	71.5 ^b^	50.0 ^c^	2.22	0.962	<0.001	0.244
Dispensable AA												
Ala	78.5 ^a^	64.8 ^abc^	71.4 ^ab^	50.3 ^cd^	78.4 ^a^	62.5 ^bc^	60.8 ^bc^	43.8 ^d^	3.62	0.173	<0.001	0.316
Asp	76.5 ^a^	34.1 ^c^	62.1 ^b^	36.4 ^c^	79.5 ^a^	33.5 ^c^	56.2 ^b^	34.9 ^c^	3.04	0.623	<0.001	0.446
Cys	69.2 ^a^	47.2 ^b^	47.6 ^b^	44.8 ^b^	73.4 ^a^	51.3 ^b^	42.1 ^b^	37.4 ^b^	3.72	0.635	<0.001	0.170
Glu	77.6 ^a^	62.8 ^bcd^	65.4 ^b^	52.4 ^cd^	83.4 ^a^	59.1 ^bcd^	62.2 ^bc^	50.0 ^d^	2.74	0.720	<0.001	0.145
Gly	81.6 ^a^	87.5 ^a^	65.5 ^ab^	52.7 ^ab^	77.6 ^a^	−23.4 ^c^	24.4 ^bc^	1.3 ^c^	11.17	0.001	<0.001	<0.001
Pro	138.1 ^a^	155.9 ^a^	111.7 ^a^	66.8 ^ab^	32.0 ^ab^	−53.3 ^ab^	−201.0 ^b^	−99.0 ^ab^	65.33	0.010	0.059	0.230
Ser	80.9 ^a^	47.1 ^c^	68.8 ^b^	53.9 ^c^	81.7 ^a^	48.1 ^c^	59.3 ^bc^	50.4 ^c^	3.02	0.277	<0.001	0.201

SEM = standard error of the means. ^a–f^ Least squares of means within a row without a common superscript letter are different (*p* < 0.05).

**Table 6:** Prediction equations for standardized ileal digestibility (SID, %) of crude protein and amino acids in commercial pigs using mini-Jeju Island native pigs (JINP) data.

Item	Regression Coefficient Parameter, %		Statistical Parameter		
	Intercept	SID in Mini-JINP	RMSE	R^2^	*p*-Value
Crude protein	−5.20	1.02	3.21	0.97	0.017
Standard error	8.88	0.13	-	-	-
*p*-Value	0.62	0.02	-	-	-
Arg	−52.95	1.54	7.33	0.85	0.080
Standard error	37.30	0.46	-	-	-
*p*-Value	0.29	0.08	-	-	-
His	−18.10	1.21	4.54	0.95	0.028
Standard error	13.94	0.21	-	-	-
*p*-Value	0.32	0.03	-	-	-
Ile	0.40	1.03	3.18	0.97	0.013
Standard error	7.70	0.12	-	-	-
*p*-Value	0.96	0.01	-	-	-
Leu	−8.21	1.13	2.60	0.98	0.012
Standard error	8.62	0.12	-	-	-
*p*-Value	0.44	0.01	-	-	-
Lys	−9.10	1.12	5.38	0.98	0.010
Standard error	5.53	0.11	-	-	-
*p*-Value	0.24	0.01	-	-	-
Met	−4.27	1.08	3.55	0.96	0.020
Standard error	10.94	0.16	-	-	-
*p*-Value	0.73	0.02	-	-	-
Phe	−6.11	1.09	3.30	0.97	0.016
Standard error	9.44	0.14	-	-	-
*p*-Value	0.58	0.02	-	-	-
Thr	18.07	0.75	8.43	0.83	0.092
Standard error	13.59	0.25	-	-	-
*p*-Value	0.31	0.09	-	-	-
Trp	11.44	0.87	6.16	0.96	0.022
Standard error	7.12	0.13	-	-	-
*p*-Value	0.25	0.02	-	-	-
Val	4.71	0.98	4.96	0.93	0.038
Standard error	12.88	0.20	-	-	-
*p*-Value	0.75	0.04	-	-	-

RMSE = root mean square error.

## Data Availability

The data presented in the current work are available.
